# Intussusception Within a Jejunostomy With Closed Loop Obstruction: A Case Report

**DOI:** 10.7759/cureus.66644

**Published:** 2024-08-11

**Authors:** Morooj ALSubhi, Abdulrahman Al Harbi, Jullanar S Alkhunein

**Affiliations:** 1 Department of Medical Imaging, King Abdulaziz Medical City, Ministry of National Guard-Heath Affairs, Riyadh, SAU; 2 Collage of Medicine, King Saud Bin Abdulaziz University for Health Sciences, Riyadh, SAU

**Keywords:** bowel resection, complications, roux-en-y gastric bypass surgery, closed loop obstruction, intussusception

## Abstract

Jejunojejunal intussusception is a rare yet severe complication of Roux-en-Y gastric bypass (RYGBP) surgery. We are presenting a unique case of retrograde jejunal intussusception with a closed-loop blockage and an associated abdominal herniation that occurred two years after a laparoscopic RYGBP. The patient presented with abdominal pain, nausea, and vomiting, prompting a clinical diagnosis and a biphasic contrast-enhanced computed tomography (CT) scan, which later revealed a complicated jejunal intussusception with signs of ischemia showing decreased wall enhancement and distal collapsed jejunal walls with complete closed-loop bowel obstruction. The case was successfully managed through emergent laparoscopy to repair the hernia and reduce the intussusception, after which the postoperative period was unremarkable. This article aims to raise awareness about this rare but significant postoperative complication and stress the importance of early medical attention in similar cases.

## Introduction

Jejunojejunal intussusception following Roux-en-Y gastric bypass surgery (RYGBP) is an uncommon but potentially fatal complication. Unlike other forms of small bowel intussusception, post-RYGBP jejunojejunal intussusception is nearly exclusively retrograde rather than antegrade in its pathogenesis. Several concepts explain the pathological progression, including jejunojejunostomy anastomosis and postoperative adhesions, Roux limb dysmotility, and bowel predisposition brought on by weight reduction [1]. 

After going through the literature review, it was revealed that abdominal discomfort, nausea, and vomiting are common signs of intussusception, which usually prompt a clinical diagnosis and computed tomography (CT) scans for verification. To elaborate, the most common diagnostic test performed for intussusception was CT scanning, which was most often preceded by plain abdominal radiographs and ultrasonography. Laparotomy was the surgical technique used primarily in management. Beyond that, revision of the jejunojejunostomy, reduction alone, or reduction with/without enteropexy were the surgical methods used to treat post-RYGB intussusception [1,2]. 

However, when comparing the prevalence of the development of intussusception with bowel obstruction, bowel obstruction with stomach discomfort is regarded as a more common postoperative complication associated with bariatric surgery, presenting with immediate and severe symptoms [1,3]. We present a case of retrograde jejunal intussusception with closed-loop obstruction that developed two years after a laparoscopic RYGBP.

## Case presentation

A 43-year-old lady with bronchial asthma came to the emergency department with sudden significant stomach discomfort that had begun the day before and was progressively worsening. Furthermore, the patient reported experiencing nausea and vomiting in conjunction with her symptoms. Upon physical examination, the patient was vitally stable and was alert, oriented, and afebrile. Her lower abdomen was soft and non-tender, with a left upper quadrant fullness initially suggesting a suspected hernia. According to the surgical history, a RYGBP took place two years ago. After the treatment, she experienced weight loss of around 60 kg from her starting body weight, bringing her current BMI down to 13. 

**Figure 1:**
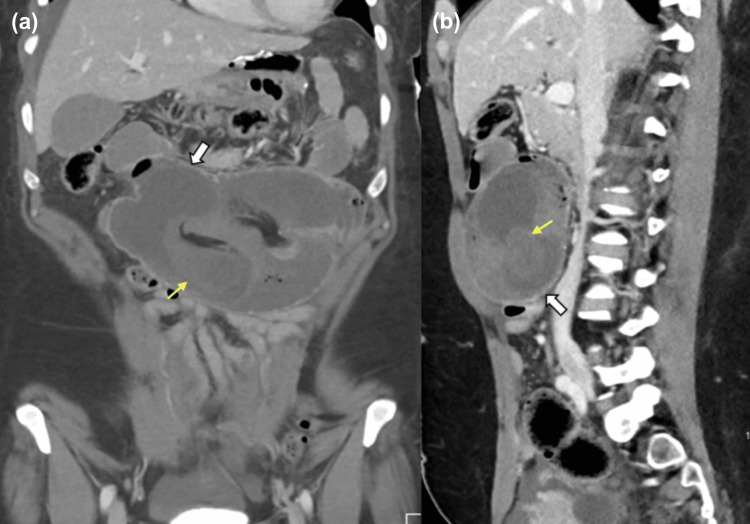
(a) Coronal and (b) sagittal reformats of an enhanced abdomen and pelvis CT scan on the portovenous phase. The Roux-en-Y afferent loop is intussuscepted within the jejunojejunostomy (white arrow). The entry point within the intussuscepted loop (yellow arrow) with mesenteric fat subsequently shows reduced enhancement of the entrapped-closed loop within the intussusception loops.

**Figure 2:**
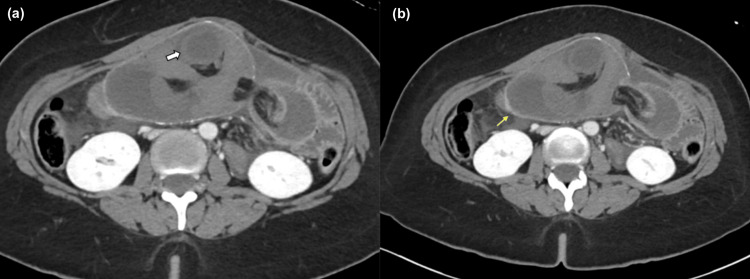
(a) Axial images show the intersecting proximal bowel loops within the intussuscepted segment with congested mucosa and reduced mucosal enhancement denoting underlying congestion (white arrow); (b) No foci of pneumatosis or signs of infarction. The jejunojejunostomy shows normal enhancement within surrounding free fluid and fat congestion (yellow arrow).

The patient underwent a biphasic contrast-enhanced CT scan of the pelvis and abdomen. It revealed a complex jejunal intussusception with complete closed-loop obstruction that was also associated with abdominal herniation. A brief portion of the jejunum encroached into the side-to-side jejunal anastomosis (Figure 1), including a mesenteric fat buildup. Additionally, it revealed thickened edematous walls and decreased enhancement, which could be concerning for ischemia. The distal jejunal loops were collapsed. There was also an associated mesenteric congestion of the intussuscepted fat, which had significant enlargement (Figure 2). The affected mesenteric vessels showed reduced enhancement without features of bowel infarction.

Urgent laparoscopic reduction of the intussusception and the repair of the Petersen hernia repair were performed via laparoscopy on the patient. Two perforations were visible in the operating room and were sutured using a Lambert double-layered stitch and a Connell method. The patient was then moved to post-anesthesia care, where the postoperative phase was uneventful with no immediate postoperative complications. The postoperative CT scan revealed a minor edematous wall jejunal thickening, a relatively small amount of ascites, and little pneumoperitoneum. Together with notable postoperative improvements, there was also subcutaneous fat stranding and emphysema over the left abdomen wall without any indications of intestinal obstruction.

However, although the mental health team was monitoring her during her hospital stay, the patient continued to have persistent tachycardia and tachypnea with critical care response team (CCRT) activation as a result of related panic attacks. After being cleared and appearing hemodynamically stable, the patient was evaluated and given instructions on when to return to the emergency room. She was then released from the hospital with a scheduled follow-up, which displayed signs of improvement with no significant postoperative complications.

## Discussion

Obesity continues to be one of the most common diseases. In Saudi Arabia, the prevalence of obesity is higher than the global average, at 35% as opposed to 13% globally [4]. For many of these people, bariatric surgery is the most effective and long-term solution to obesity. It is frequently recommended for severely obese people who have not responded well to conventional weight loss techniques [5]. Bariatric surgery has contributed to long-term weight loss and the treatment of obesity-related comorbidities, leading to a dramatic increase in the number of bariatric procedures undertaken [2]. 

Various bariatric surgeries such as laparoscopic adjustable gastric banding, laparoscopic sleeve gastrectomy, and laparoscopic RYGBP are available. Reducing body weight and enhancing quality of life are the main goals of the RYGBP operation, a standard procedure in Saudi Arabia. However, various major and minor surgical complications can arise from the RYGBP operation. Major complications could include bleeding, gastrointestinal leaks, and internal herniations. Minor side effects may include nausea, vomiting, and dysphagia. Nonetheless, RYGBP surgery puts patients at risk for serious long-term side effects such as small intestinal obstruction [2,6]. 

It is estimated that 0.5-5% of patients experience intestinal obstruction following RYGBP. Internal hernia and adhesions are the most common causes of intestinal obstruction following RYGBP [1,3]. In addition, 0.07-0.6% of cases of small bowel intussusception occur following RYGBP; this is a rare but potentially serious cause of obstruction [2,7]. Although it is uncommon for bowel obstruction and intussusception to follow RYGBP, Aregawi et al. reported a case of closed-loop bowel obstruction within an isolated small bowel intussusception [8].

The pathogenesis of intussusception, which is virtually entirely retrograde in post-RYGB patients, is yet unknown. In the general adult population, intussusception is typically antegrade. One prominent theory has been that bowel dysmotility is the primary reason [1]. Retrograde intussusception is also typically observed in female individuals who have undergone significant weight reduction. Another theory is that the fixed mesentery of the bypassed jejunum becomes hypermobile as a consequence of mesenteric fat loss [9]. Some research indicates that individuals, like our patient, who experience an interval decline in BMI of 15.8 kg/m^2^ are more inclined to develop retrograde intussusception [10].

Intussusception can manifest clinically as excruciating stomach discomfort, nausea, and vomiting. The extent of the intussusception and the existence of ischemia or necrosis may determine the precise surgical technique needed to relieve the obstruction, which is frequently the case. Medical providers must understand that intussusception could potentially be the reason behind such clinical presentation in people with RYGB history [2]. In cases that have been documented, the median time for presentation was 36 months after surgery [11], as opposed to our patient, who experienced an incidence around 24 months after surgery. 

As in our case, intussusception combined with closed-loop bowel obstruction is a surgical emergency that calls for immediate consultation with a bariatric surgeon. Given the rarity and severity of the combined presentation of intussusception and closed-loop obstruction following RYGB, it can be challenging to identify the unique risk variables that might raise the likelihood of such a presentation [12]. This study can add more clarity to the approach to patients with similar presentations and past surgical history. 

## Conclusions

Clinicians should be highly cautious when approaching patients who have a positive surgical history of RYGBP surgery and who are symptomatically presenting with signs of small bowel obstruction due to the recent increase in its prevalence and the uncommon late complication of retrograde intussusception, as it can cause a delay in the diagnosis and management.
